# Supplementary material for: A Genome-Wide View of Transcriptional Responses during Aphis glycines Infestation in Soybean
Source: Int J Mol Sci. 2020 Jul 22;21(15):5191. doi: 10.3390/ijms21155191 (PMC7432633; doi:10.3390/ijms21155191)
Supplement: Supplementary file 1 [file ijms-21-05191-s001.zip › Supplementary Materials/Figure S1.pdf]

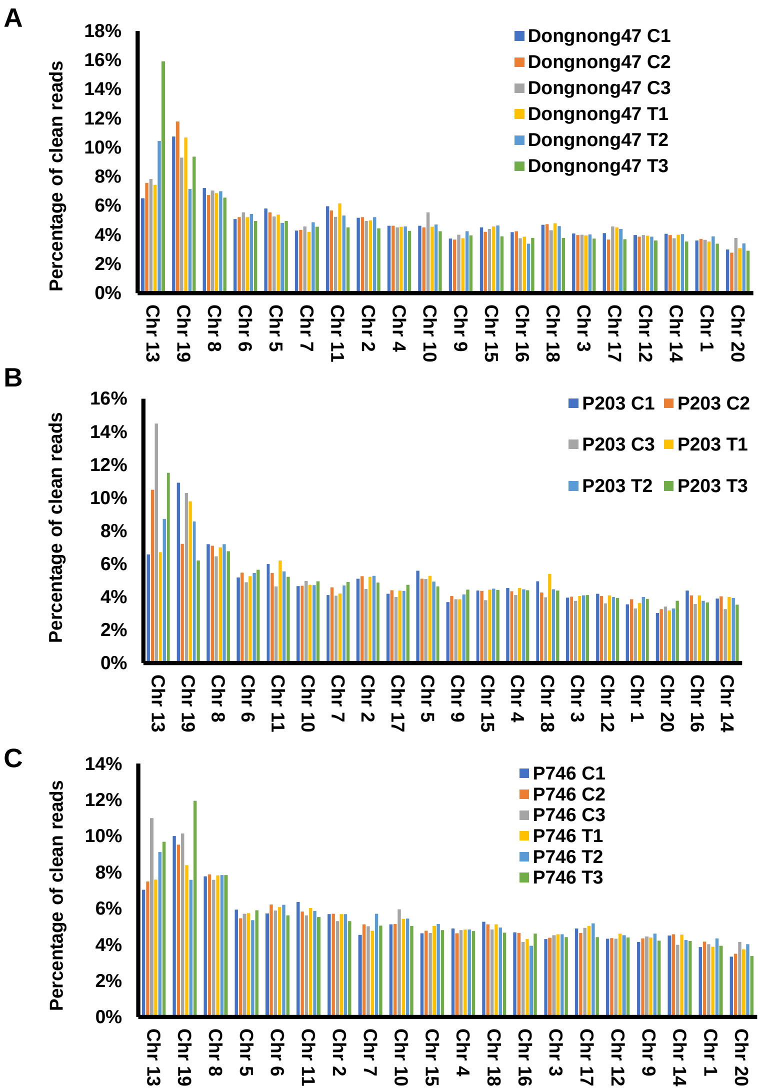

Figure S1 Distribution of clean reads on chromosomes in soybean cultivars Dongnong47 (A), P203 (B), and P746 (C). C1, mock treatment at 24 h after infestation (HAI); C2, mock treatment at 48 HAI; C3, mock treatment at 96 HAI; T1, aphid infestation at 24 HAI; T2, aphid infestation at 48 HAI; T3, aphid infestation at 96 HAI.
