# Supplementary material for: A Genome-Wide View of Transcriptional Responses during Aphis glycines Infestation in Soybean
Source: Int J Mol Sci. 2020 Jul 22;21(15):5191. doi: 10.3390/ijms21155191 (PMC7432633; doi:10.3390/ijms21155191)
Supplement: Supplementary file 1 [file ijms-21-05191-s001.zip › Supplementary Materials/Figure S2.pdf]

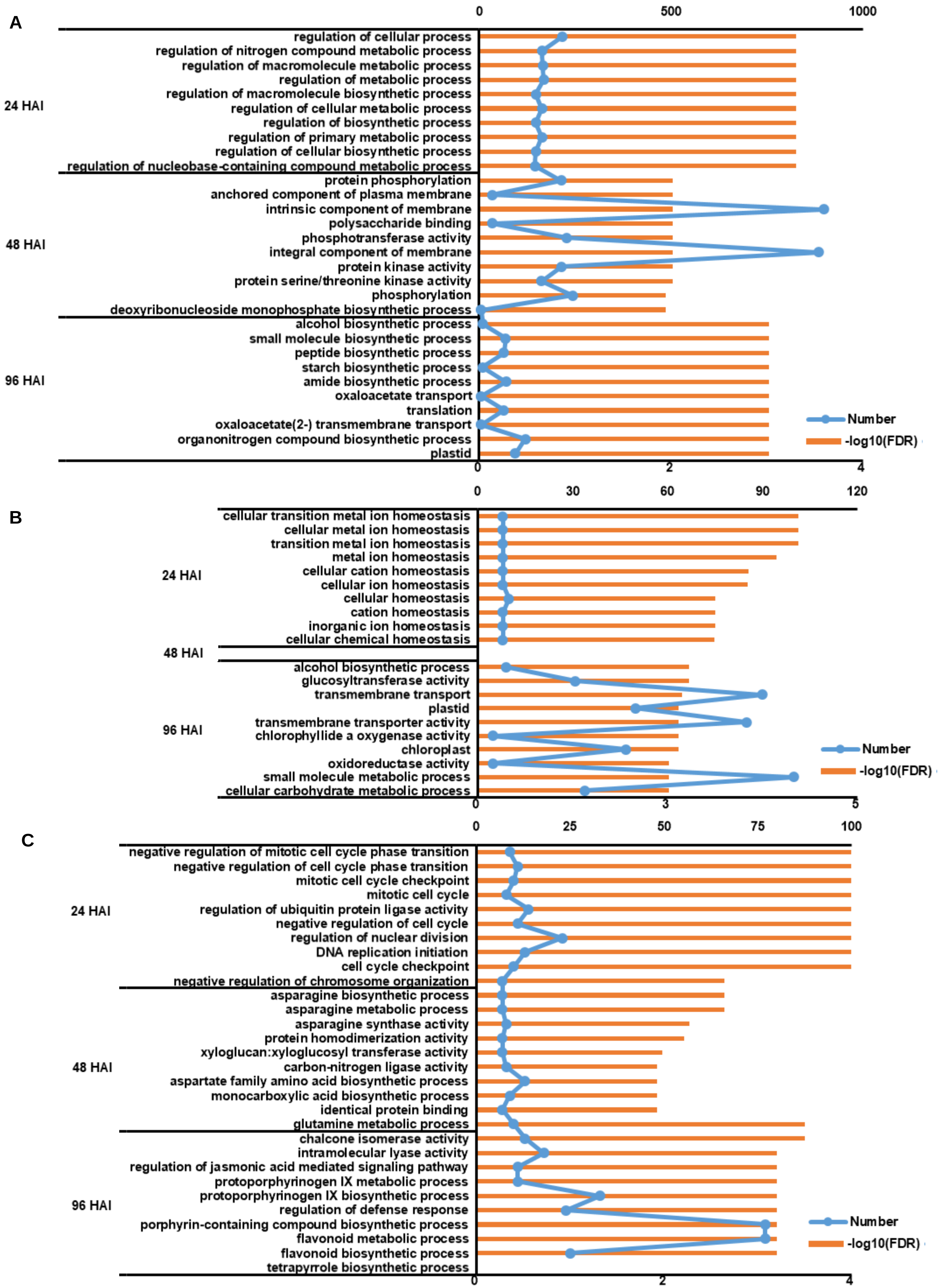

Figure S2 Top 10 significantly enriched (FDR<0.05) GO terms in Dongnong47 (D47) (A), P203 (B) and P746 (C) at 24, 48 and 96 HAI respectively.
