# Supplementary material for: A Genome-Wide View of Transcriptional Responses during Aphis glycines Infestation in Soybean
Source: Int J Mol Sci. 2020 Jul 22;21(15):5191. doi: 10.3390/ijms21155191 (PMC7432633; doi:10.3390/ijms21155191)
Supplement: Supplementary file 1 [file ijms-21-05191-s001.zip › Supplementary Materials/Figure S4.pdf]

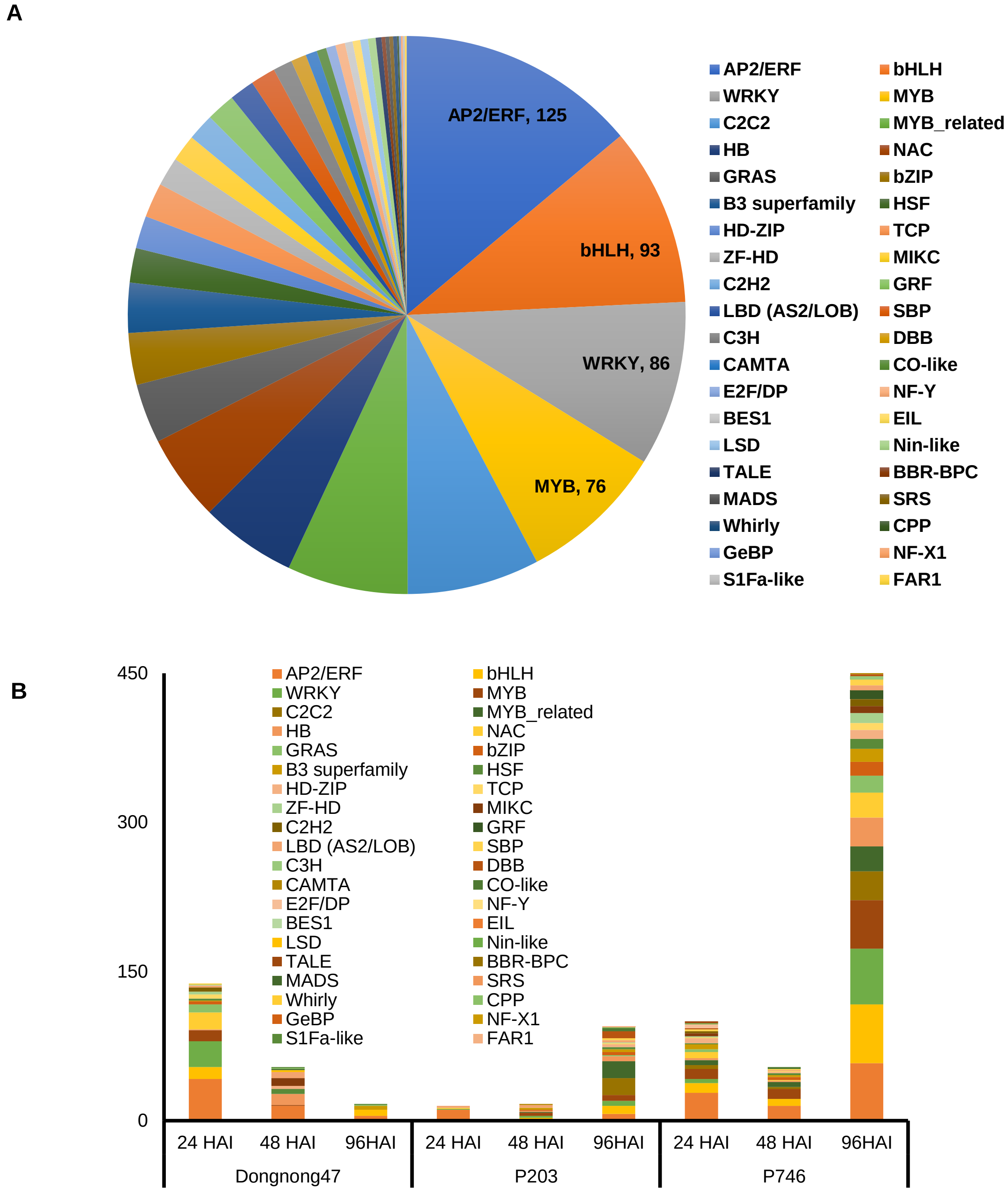

Figure S4 Distribution of different expressed transcription factors (TF). (A) Numbers of TFs in each families. (B) Differential expressed in all genotypes
